# Clinical effect of a modified superficial temporal artery-middle cerebral artery bypass surgery in Moyamoya disease treatment

**DOI:** 10.3389/fneur.2023.1273822

**Published:** 2023-10-24

**Authors:** Liang Lu, Yimin Huang, Yang Han, Yu Li, Xueyan Wan, Juan Chen, Xincheng Zhang, Kai Shu, Ting Lei, Sheng Wang, Chao Gan, Huaqiu Zhang

**Affiliations:** Department of Neurosurgery, Tongji Hospital, Tongji Medical College, Huazhong University of Science and Technology, Wuhan, China

**Keywords:** Moyamoya disease, STA-MCA bypass, cerebral revascularization, modified surgical technique, vascular disorders

## Abstract

**Background:**

Cerebral extracranial-intracranial (EC-IC) revascularization technique (superficial temporal artery-middle cerebral artery (STA-MCA) bypass grafting) has become the preferred surgical method for the treatment of Moyamoya disease (MMD). We attempted to completely free the two branches of the superficial temporal artery without disconnection. Extracranial and intracranial blood flow reconstruction were then modified by selectively performing a direct bypass technique on one branch and a patch fusion technique on the other of the STA based on the blood flow and the vascular diameter of the intracranial surface blood vessels.

**Methods:**

A series of modified STA-MCA bypass surgeries performed consecutively between March 2022 and March 2023 were reviewed and compared to conventional combined bypass surgeries performed during the same period. The following information was collected from all enrolled patients: demographic characteristics, clinical symptoms, and preoperative and postoperative imaging, including Suzuki stage and Matsushima grade. The modified Rankin scale (mRS) was used to assess the changes in neurological status before and after surgery.

**Results:**

A total of 41 patients with Moyamoya disease (MMD) who underwent cerebral revascularization were included in this study, of which 30 were conventional revascularization and 11 were modified revascularization. The mean age was 49.91 years, and 18 (43.9%) of the patients were women. The modified group had a lower incidence of cerebral hyperperfusion syndrome (18.2%) than the conventional group (23.3%). After at least 3 months of follow-up, the bypass patency rate remained 100% in the modified group and 93.3% in the conventional group. All patients in the modified group achieved a better Matsushima grade (A + B), with six (54.5%) having an A and five (45.5%) having a B. In contrast, four patients (13.3%) in the conventional group had a Matsushima grade of C. In all, 72.8% of the modified group had postoperative mRS scores of 0 and 1, which was higher than that of the traditional group (63.3%).

**Conclusion:**

The improved STA-MCA bypass could provide blood flow to multiple cerebral ischemic areas, reduce excessive blood perfusion, and ensure blood supply to the scalp, with lower complications and better clinical benefits than the traditional combined bypass.

## Introduction

In recent years, with the improvement of clinical diagnosis and treatment and the popularization of intracranial angiography, the incidence and diagnosis of Moyamoya disease and Moyamoya syndrome have been increasing rapidly in China (1). Currently, extracranial-intracranial blood flow reconstruction techniques (superficial temporal-middle cerebral artery bypass grafting plus Encephalo-myo-synangiosis) have become the preferred surgical option for the treatment of Moyamoya disease and have achieved good results (2, 3). However, due to the mismatch in thickness and blood flow between intracranial recipient vessels (middle cerebral artery M3 and M4) and extracranial donor vessels (superficial temporal artery frontal or parietal branches), the success rate of direct vascular anastomosis and surgical outcome are often affected (4). Especially when the superficial temporal artery branch is significantly thicker or the blood flow is too large, the direct single-vessel end-side anastomosis may lead to over-perfusion of blood flow in the recipient vessel, which may cause brain tissue edema and neurological damage, or even hemorrhage and infarction, endangering the patient’s life and safety (5, 6). To address this situation, clinicians often use a variety of means to reduce donor blood flow and flow rate by narrowing the lumen of the donor vessel or increasing the degree of vessel turning (7). Some centers have also proposed the use of lateral anastomosis to reduce the donor blood flow by shunting part of the blood flow into the skull while preserving the original superficial temporal artery branch flow path (8). In recent years, our center has found that by completely freeing the superficial temporal artery and its two branches, and then selectively adopting one direct bypass and one patch fusion technique according to the branches and intracranial cerebral surface vessels, we can avoid excessive shunting from the outside to the inside of the skull, ensure the blood supply to the scalp, and add a pathway to reconstruct the intracranial and extracranial blood flow through the fusion of blood vessels, which can be said to be killing three birds with one stone. In the present study, 41 cases of patients with Moyamoya disease have been treated with extracranial-intracranial blood flow reconstruction, and the demographic characteristics, surgical methods, and clinical effects are reported in order to obtain a preliminary result about this modified bypass strategy.

## Materials and methods

### Study population and design

We retrospectively evaluated patients with MMD who underwent bypass surgery from March 2022 to March 2023 at Tongji Hospital and who were treated by the same group. The inclusion criteria were as follows: (1) patients with MMD confirmed by digital subtraction angiography (DSA) or magnetic resonance angiography (MRA) according to previous reports (2), (2) patients older than 18 years old, and (3) patients who underwent surgical therapy. The exclusion criteria were as follows: (1) patients who did not undergo follow-up evaluation, (2) patients who underwent only encephalo-duro-arterio-synangiosis (EDAS) or direct bypass without encephalo-myo-synangiosis (EMS), and (3) patients with secondary Moyamoya syndrome caused by other reasons, such as autoimmune diseases, Down’s syndrome, and radiation exposure to the head. The following information was collected from all enrolled patients: demographic characteristics, preoperative and postoperative imaging, clinical symptoms, surgical method, complications, and follow-up. The study was approved by the Ethics Committee of Tongji Hospital. Due to the retrospective nature of the cohort study, the need for informed consent was waived.

### Surgical technique

**Figure 1:**
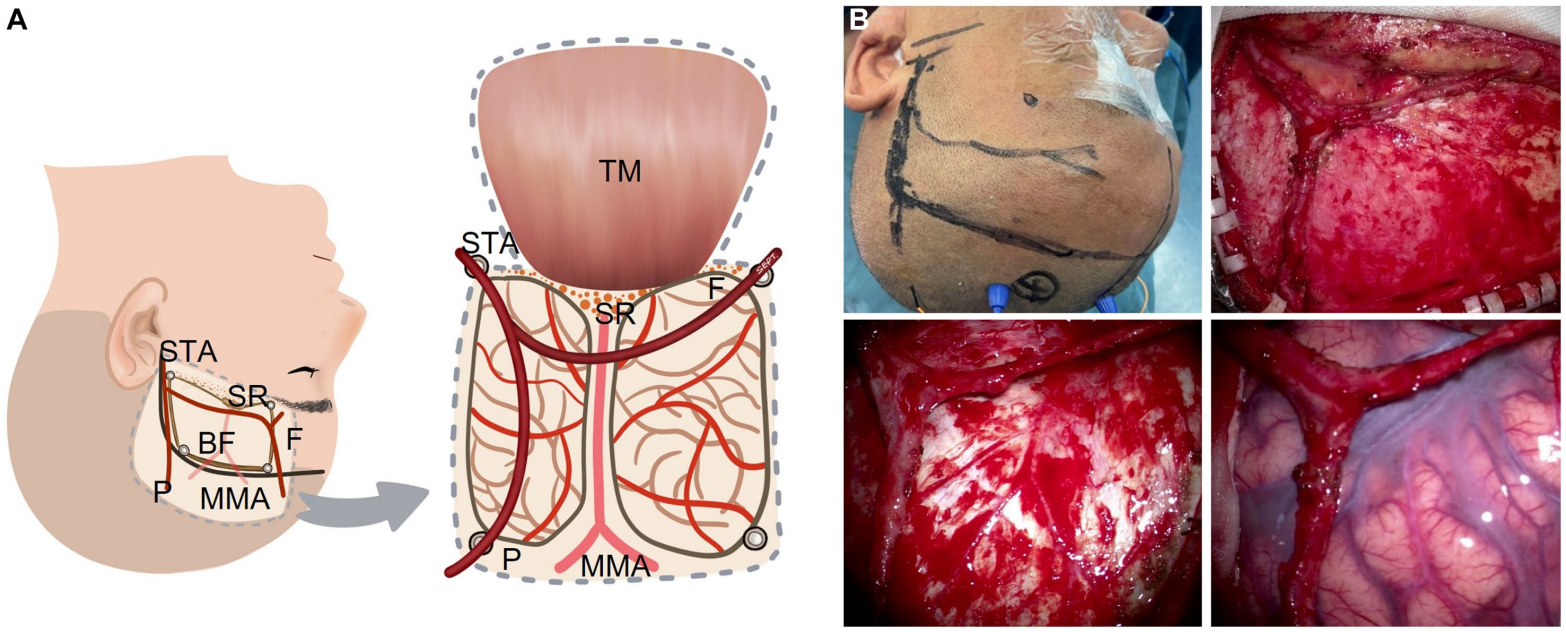
STA dissecting procedure in a modified bypass surgery with both branches of the STA intact without a cut. **(A)** Illustration (artist rendition, left hemisphere). **(B)** A representative patient. BF: bone flap; F: frontal branch of the STA; MMA: middle meningeal artery; P: parietal branch of the STA; SR: sphenoid ridge; STA: superficial temporary artery; TM: temporal muscle.

**Figure 2:**
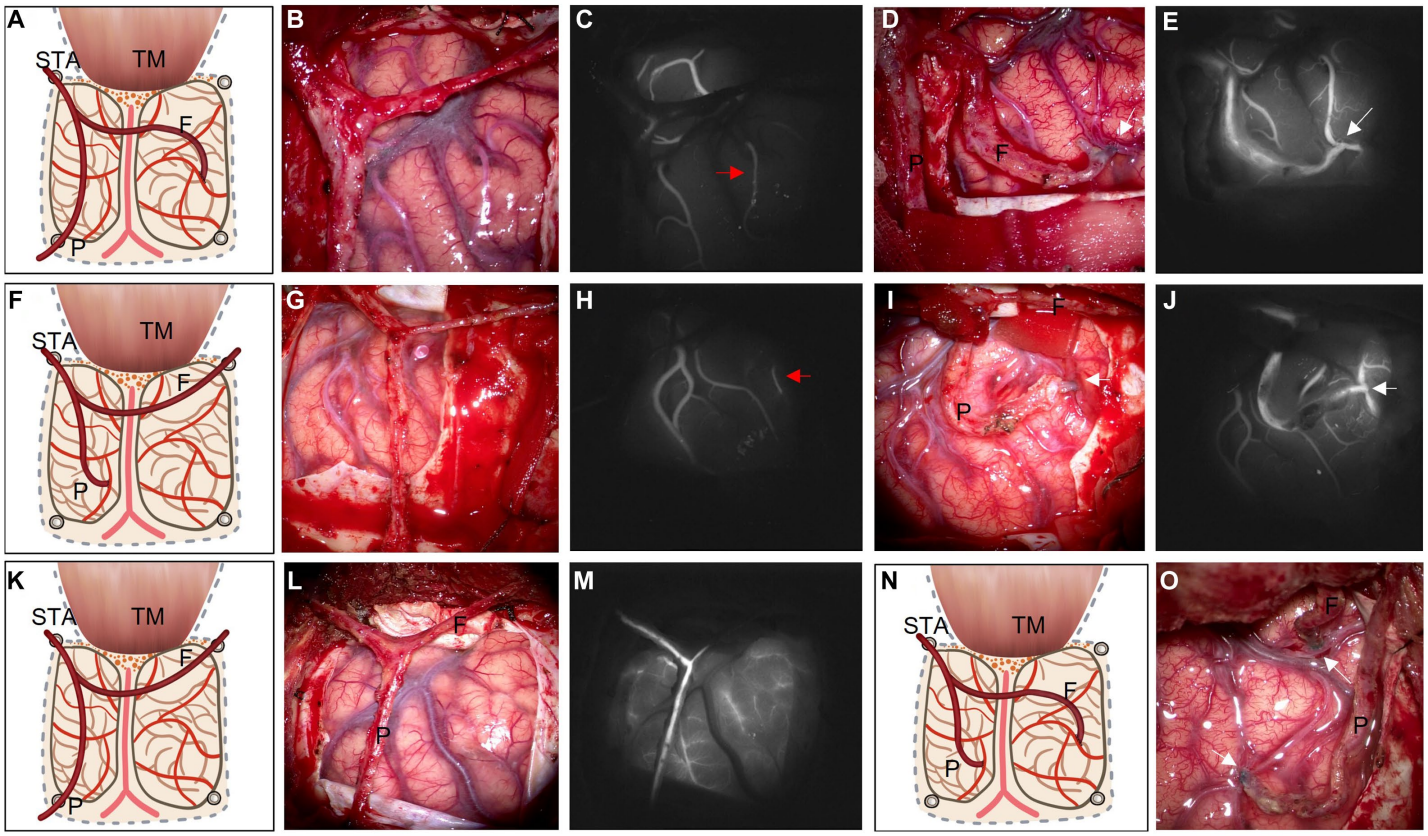
Based on ischemic area and ICG videoangiography, illustrations of four types of operative strategies are listed as follows: 1. frontal branch for STA-MCA end-to-side bypass with parietal branch EDAMS **(A)**; 2. parietal branch for STA-MCA bypass with frontal branch EDAMS **(F)**; 3. EDAMS with both branches of the STA without a cut **(K)**; 4. Double-barrel bypass with both branches of the STA **(N)**. After the STA is dissected without a cut **(B,G,L)**, ICG videoangiography is used to evaluate the relatively slow-flow recipient M4 (**C,H**, red arrow). Sometimes, recipient M4 cannot be found **(M)**. After the bypass is finished (**D,I,O**, white arrow indicate the anastomosis point), ICG videoangiography is used again to evaluate the patency of the bypass vessel (**E,J**, white arrow). F: frontal branch of the STA; P: parietal branch of the STA; STA: superficial temporary artery; TM: temporal muscle.

The conventional surgical techniques were described in previous research (2, 4). The modified bypass scheme of STA dissection is shown in Figure 1 and four representative surgical methods are shown in Figure 2. In conventional surgery, a single STA branch was traditionally used as a donor vessel for STA-MCA anastomosis combined with EMS. The parietal branch of the STA was usually used since the frontal branch could form a collateral formation naturally. The middle meningeal artery (MMA) was preserved as much as possible. When the recipient vessel was not found in the brain cortex, an EDAMS procedure was used with the frontal branch. When the STA could not be used, the cortex was covered with muscle alone with EMS. In modified surgery, direct anastomosis combined with indirect synangiosis is performed to use all STA vessels and tissues capable of providing future collateral formation. Both the frontal and parietal branches are utilized.

Before the scalp incision is made, the course of the STA (especially the parietal branch) is confirmed with touch pulsation or Doppler ultrasound probe. Then the scalp incision is made along the course of the parietal branch of the STA and from the midway point the incision is turned around nearly 90 degrees anteriorly to the midline. Both the frontal and parietal branches of the STA are adequately dissected and secured without cutoff. The temporal muscle is one of the sources of indirect synangiosis (EMS). As the dural opening is covered by the temporal muscle, we separate the temporal muscle as widely as possible. We usually make four burr holes for craniotomy. Of the four burr holes, one is placed just proximal to the STA, one at the distal end of the parietal branch, and one at the distal end of the frontal branch located close to the keyhole. The last one is placed at the superior temporal line where the temporal muscle is attached. In order to preserve the MMA, which is easily injured if the incision is made at the base of the temporal bone, we make the temporal bone thinner using a drill and remove the bone piece by piece during the craniotomy. A window-like incision is made in the dura mater without cutting the main branch of the MMA. The dura opening should be as wide as possible.

Before STA-MCA anastomosis was undertaken, several details needed to be cleared. Based on the results of the cerebral blood flow (CBF) study and indocyanine green (ICG) video angiography, ischemic regions and alternative recipient vessels were identified. We empirically preferred to choose the slow-flow vessel under ICG video angiography in the low-perfusion area as the recipient vessel if possible (Figures 2C,H, red arrow). The selection of a donor vessel should also consider compensation, vessel diameter, ischemic zone, and so on. When intracranial compensation exists in one branch of the STA, the other one is selected as the donor vessel. Select a vessel that matches the recipient vessel as the donor vessel. Referring to the ischemic area factor, bypass strategies are as follows: (1) When low-perfusion is mainly located in the frontal area, the frontal branch is preferred as the donor vessel for STA-MCA anastomosis, and the parietal branch is placed on the surface of the exposed cortex; (2) If a preoperative CBF study reveals a low-perfusion area mainly at the parietal area, then the parietal branch is chosen as the donor vessel; and (3) If no recipient vessel can be found, both branches of STA are performed for indirect revascularization. After STA-MCA anastomosis, the largest possible area of the exposed cortex is covered with temporal muscle and another branch of STA (EDAMS).

### Radiological and clinical evaluation

All patients underwent preoperative and postoperative DSA/MRA and CT perfusion (CTP)/arterial spin labeling (ASL). MRA or vascular DSA was used preoperatively to confirm stenosis and to determine the Suzuki stage in patients with MMD (9), and postoperatively to assess bypass patency and to record Matsushima grade to assess revascularization (10). CTP or ASL was used preoperatively to identify the ischemic area in patients with MMD and to select the surgical hemisphere and the area for revascularization accordingly, and postoperatively to evaluate the alteration of perfusion. The Suzuki stage was recorded preoperatively to assess the severity of MMD patients. Specifically, in Matsushima grade, “A” represents hemodialysis in 2/3 of the middle cerebral artery (MCA) distribution, “B” represents hemodialysis in between 1/3 and 2/3 of the MCA distribution, and “C” represents poor or no hemodialysis. Patients’ preoperative clinical symptoms and postoperative complications were recorded. The modified Rankin scale (mRS) was used to assess the changes in patients’ neurologic functional status before and after surgery (11).

### Statistical analysis

Statistical analyses were performed using R software (version 3.6.3), with categorical data being expressed as percentages and continuous variables being expressed as mean ± standard deviation (SD). The Student’s t-test and Wilcoxon test were used to compare the two continuous variables and the Chi-square test, Yates’ correction, or Fisher’s exact tests were used for categorical variables. *p* < 0.05 was considered a statistically significant difference.

## Results

### Patient characteristics

A total of 41 patients with Moyamoya disease who underwent cerebral revascularization were included in this study (Table 1), of which 30 underwent conventional revascularization and 11 underwent modified revascularization. The mean age was 49.91 years, and 18 (43.9%) patients were women. There were no significant differences between the traditional and modified groups in terms of sex, age, symptoms, and comorbidities. In the traditional group, 14 cases of the right hemisphere and 16 cases of the left hemisphere underwent cerebral revascularization, and in the modified group, 4 cases of the right hemisphere and 7 cases of the left hemisphere underwent cerebral revascularization.

**Table 1:** Characteristics of patients with Moyamoya disease (MMD).

Characteristic	All (*n* = 41)	Traditional (*n* = 30)	Modified (*n* = 11)	*p*	Method
Age, years	51.0 (43.5,56.0)	51.0 (43.0,56.0)	52.0 (44.0,57.0)	0.689	Wilcoxon test
Sex				1.000	Chi-square
Female	18 (43.9%)	13 (43.3%)	5 (45.5%)		
Male	23 (56.1%)	17 (56.7%)	6 (54.4%)		
Follow-up, months	3 (3, 6)	3 (3, 6)	3 (3, 5)		Wilcoxon test
Hemisphere				0.815	Yates’ correction
Rt	18 (43.9%)	14 (46.7%)	4 (36.4%)		
Lt	23 (56.1%)	16 (53.3%)	7 (63.6%)		
Symptom				0.850	Yates’ correction
Headache and Dizziness	17 (41.5%)	13 (43.3%)	4 (36.4%)		
Limb weakness	19 (46.3%)	14 (46.7%)	5 (45.5%)		
Aphasia	6 (14.6%)	4 (13.3%)	2 (18.2%)		
Intellectual disability	3 (7.3%)	2 (6.7%)	1 (9.1%)		
Blurred vision	4 (9.8%)	3 (10.0%)	1 (9.1%)		
Transient disorders of consciousness	5 (12.2%)	4 (13.3%)	1 (9.1%)		
Presenting symptom				0.936	Yates’ correction
Hemorrhage	9 (22.0%)	7 (23.3%)	2 (18.2%)		
Infarction	15 (36.6%)	10 (33.3%)	5 (45.5%)		
TIA	5 (12.2%)	4 (13.3%)	1 (9.1%)		
Chronic cerebral ischemia	12 (29.3%)	9 (30.0%)	3 (27.3%)		
Comorbidities					
Hypertention	14 (34.1%)	10 (33.3%)	4 (36.4%)	1.000	Yates’ correction
Diabetes mellitus	7 (17.1%)	6 (20.0%)	1 (9.1%)	0.723	Yates’ correction
Hyperlipidemia	7 (17.1%)	5 (16.7%)	2 (18.2%)	1.000	Yates’ correction
Surgical method				0.260	Yates’ correction
Single-bypass+EMS/EDAMS	33 (80.5%)	25 (83.3%)	8 (72.7%)		
Double-barrel bypass	1 (2.4%)	0 (0.0%)	1 (9.1%)		
EDAMS	5 (12.2%)	3 (10.0%)	2 (18.2%)		
EMS	2 (4.9%)	2 (6.7%)	0 (0.0%)		
Suzuki stage				0.984	Yates’ correction
II	4 (9.8%)	3 (10.0%)	1 (9.1%)		
III	18 (43.9%)	13 (43.3%)	5 (45.5%)		
IV	14 (34.1%)	10 (33.3%)	4 (36.4%)		
V	5 (12.2%)	4 (13.3%)	1 (9.1%)		
mRS score preop				0.947	Yates’ correction
0	1 (2.4%)	1 (3.3%)	0 (0.0%)		
1	16 (39.0%)	11 (36.7%)	5 (45.5%)		
2	16 (39.0%)	12 (40.0%)	4 (36.4%)		
3	5 (12.2%)	4 (13.3%)	1 (9.1%)		
4	3 (7.3%)	2 (6.7%)	1 (9.1%)		

TIA, transient ischemic attack; EMS, encephalo-myo-synangiosis; EDAMS, encephalo-duro-arterio-myo-synangiosis; mRS, the modified Rankin scale.

The starting symptoms, from most to least, were cerebral infarction (36.6%), chronic cerebral ischemia (29.3%), cerebral hemorrhage (22.0%), and TIA (12.2%). Specifically, headache, dizziness (41.5%), and limb weakness (46.3%) were more prevalent, while aphasia (14.6%), intellectual disability (7.3%), blurred vision (9.8%), and transient impairment of consciousness (12.2%) were relatively rare. As for vascular diseases, some patients had a combination of hypertension (34.1%), diabetes mellitus (17.1%), and hyperlipidemia (17.1%).

### Preoperative imaging and functional analysis

All patients underwent preoperative cerebral angiography or MRA to assess and analyze the morphology of the cerebral vessels, and the Suzuki stage was used to classify patients with MMD. The vast majority of patients (Table 1) were in grade III (43.9%) or IV (34.1%), and a small number were in grade II (9.8%) or V (12.2%). The present study did not contain patients in grades I and VI. The functional status of the patients was then assessed using the mRS, with the majority of the patients located in grade 1 (39.0%) or 2 (39.0%), and a small proportion in grade 0 (2.4%), 3 (12.2%), or 4 (7.3%); the present study did not contain patients in grades 5 and 6. The majority of patients (80.5%) underwent improved single bypass combined with EMS/EDAMS. One patient underwent a double-barrel bypass due to multi-regional ischemia and permitting conditions. Five patients (12.2%) and two patients (4.9%) underwent EDAMS and EMS, respectively.

### Postoperative complications

There was no significant difference in the incidence of postoperative complications between the conventional and modified groups (Table 2), which may be related to the smaller number of cases. Even so, we were able to observe that the modified group had a lower incidence of cerebral hyperperfusion syndrome (18.2%) than the conventional group (23.3%), with complications such as myasthenia gravis (3.3%) and epilepsy (3.3%) in some of the patients in the conventional group but not in those in the modified group. Similarly, in terms of scalp-related complications, some patients in the conventional group had poor scalp healing (3.3%) and subcutaneous effusion (6.7%), whereas patients in the modified group did not.

**Table 2:** Postoperative complications.

Characteristic	Traditional (*n* = 30)	Modified (*n* = 11)	*p*	Method
Cerebral infarction	2 (6.7%)	1 (9.1%)	1.000	Yates’ correction
Cerebral hemorrhage	1 (3.3%)	0 (0.0%)	1.000	Fisher’s exact test
Cerebral hyperperfusion syndrome	7 (23.3%)	2 (18.2%)	1.000	Yates’ correction
Aphasia	2 (6.7%)	1 (9.1%)		
Myasthenia	1 (3.3%)	0 (0.0%)		
Epilepsy	1 (3.3%)	0 (0.0%)		
Vertigo and Vomiting	3 (10.0%)	1 (9.1%)		
Disturbance of consciousness	0 (0.0%)	0 (0.0%)		
Scalp	3 (10.0%)	0 (0.0%)	0.680	Fisher’s exact test
Poor scalp healing	1 (3.3%)	0 (0.0%)		
Subcutaneous effusion	2 (6.7%)	0 (0.0%)		

### Postoperative outcome

Bypass patency during surgery was 100% in both the conventional and modified groups (Table 3). After at least 3 months of follow-up, the bypass patency rate remained 100% in the modified group and decreased to 93.3% in the conventional group, of which the two patients with bypass obstruction were patients who underwent conventional indirect bypass surgery (Supplementary Table S1).

**Table 3:** Postoperative outcome.

Characteristic	Traditional (*n* = 30)	Modified (*n* = 11)	*p*	Method
Bypass patency during OP	30 (100.0%)	11 (100.0%)	1.000	Fisher’s exact test
Bypass patency at last FU	28 (93.3%)	11 (100.0%)	0.952	Fisher’s exact test
Matsushima grade at the last FU			0.346	Yates’ correction
A	11 (36.7%)	6 (54.5%)		
B	15 (50.0%)	5 (45.5%)		
C	4 (13.3%)	0 (0.0%)		
mRS score at the last FU			0.947	Yates’ correction
0	9 (30.0%)	4 (36.4%)		
1	10 (33.3%)	4 (36.4%)		
2	8 (26.7%)	2 (18.2%)		
3	3 (10.0%)	1 (9.1%)		
4	0 (0.0%)	0 (0.0%)		

FU, follow-up; OP, operation.

**Figure 3:**
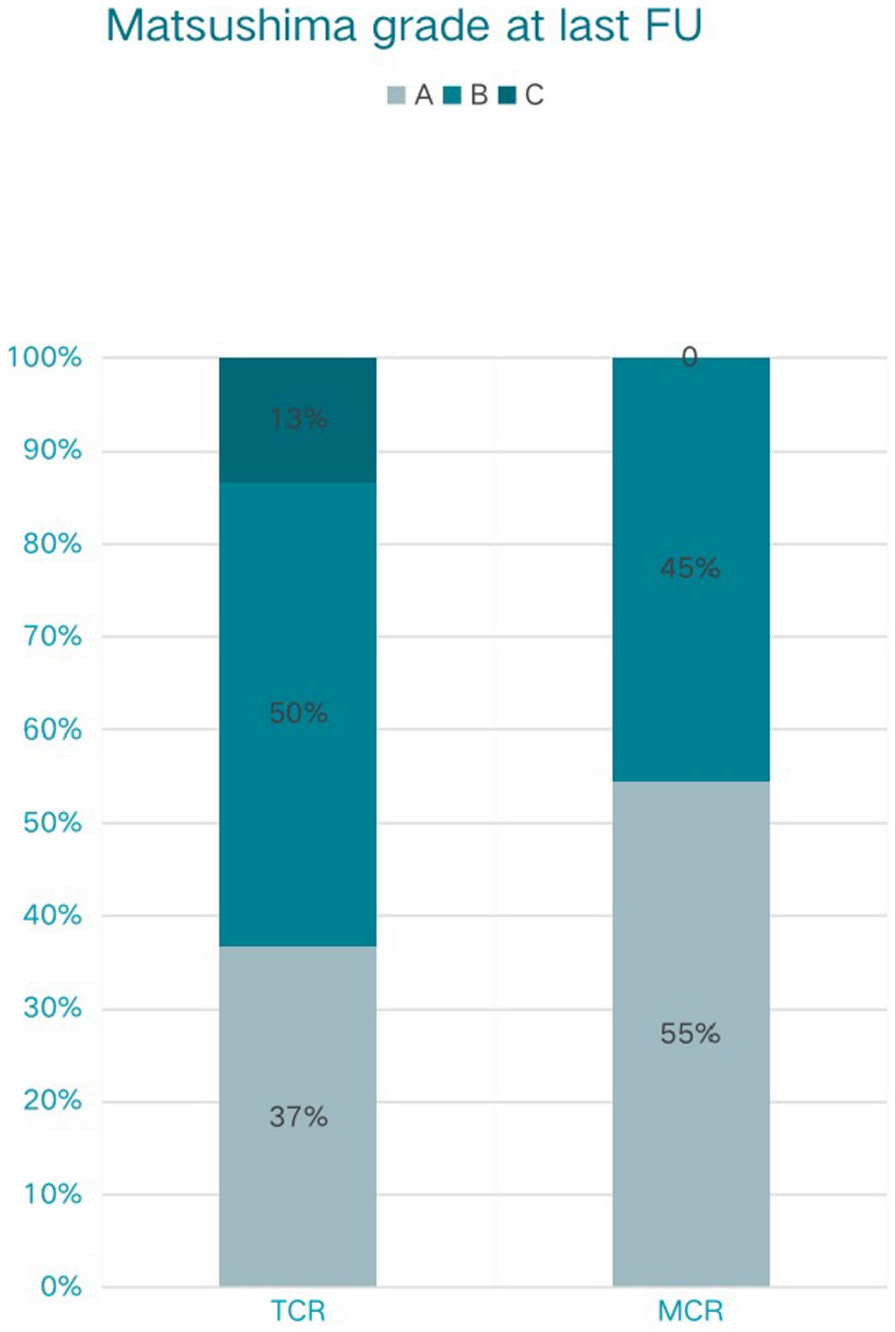
Matsushima grade at last follow-up (FU) of traditional cerebral revascularization (TCR) and modified cerebral revascularization (MCR).

**Figure 4:**
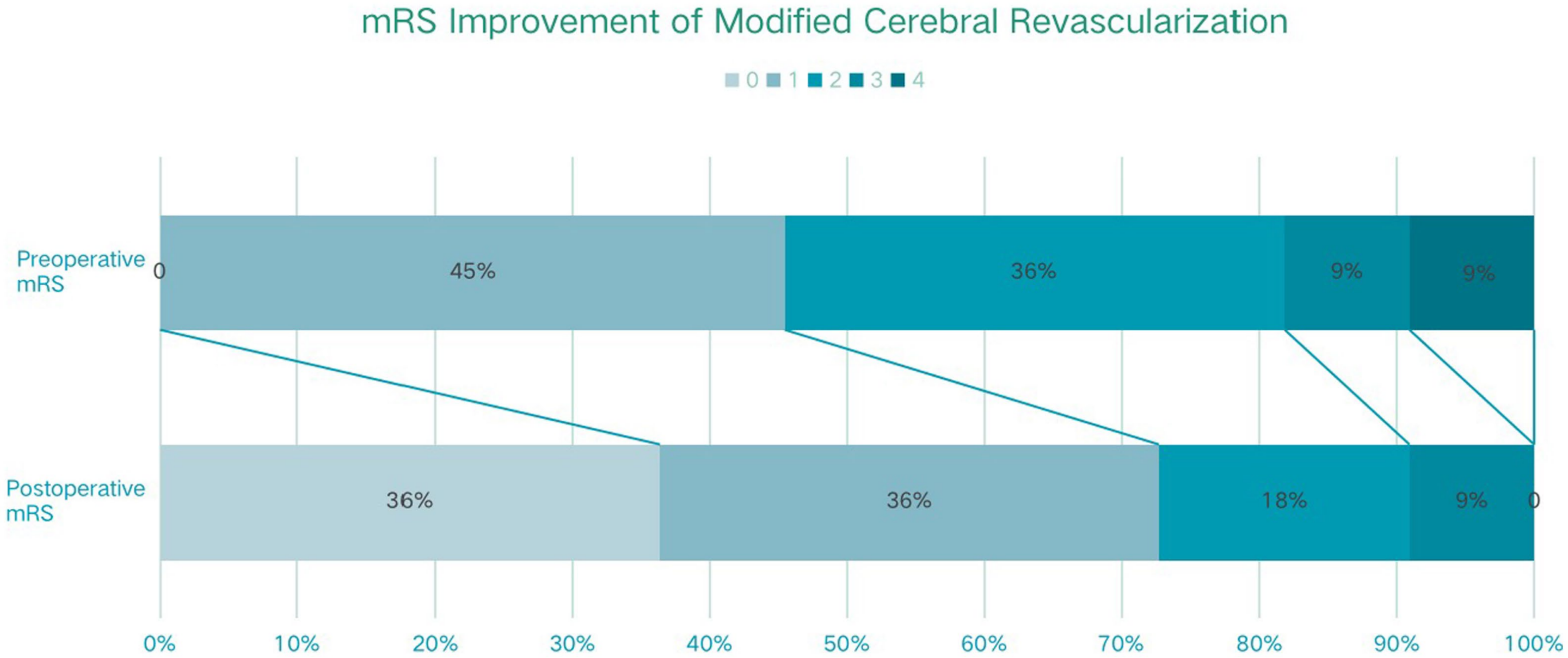
The modified Rankin scale (mRS) improvement of traditional cerebral revascularization (TCR) and modified cerebral revascularization (MCR).

All patients in the modified group achieved a better Matsushima grade (A + B) (Figure 3), with six (54.5%) having an A and five (45.5%) having a B. In contrast, 4 patients (13.3%) in the conventional group had a Matsushima grade of C, i.e., poor revascularization, and 11 patients in the conventional group had an A in their rating (36.7%). The preoperative and postoperative mRS changes reflected the impact of surgery on patients’ neurological recovery (Figure 4), and both groups achieved a good improvement in mRS scores, with 72.8% of the modified group having postoperative mRS scores of 0 and 1, which was higher than that of the traditional group (63.3%). Among the patients who underwent indirect bypass surgery, the postoperative mRS score of the modified group was higher than that of the traditional group (*p* = 0.099) (Supplementary Table S1), but due to the small amount of data, it was necessary to further expand the sample size for verification.

## Discussion

Our present study discusses a modified surgical approach to extracranial-intracranial blood flow reconstruction for the treatment of Moyamoya disease (or Moyamoya syndrome). To achieve the lowest complications and the best clinical benefit, we introduced a modified surgical method by completely separating both branches of the superficial temporal artery and selectively performing different bypasses depending on the development of the patient’s middle cerebral artery (M4) branches.

Anastomosis through the superficial temporal artery branches and the cortical branches of the middle cerebral artery (direct bypass technique) combined with temporalis muscle-dural-fine-mold apposition (indirect bypass technique) has long been the preferred means for the treatment of Moyamoya disease (2, 12, 13). However, hyperperfusion or hypoperfusion of blood flow due to donor-recipient vascular mismatch and secondary cerebral edema, infarction, or hemorrhage are important factors that seriously affect the safety and efficacy of the procedure (14, 15). The present modified bypass surgical approach is performed by completely freeing the superficial temporal artery and its two branches, and then selectively choosing the bypass method based on the degree of matching between the recipient and donor vessels. In the case of the superficial temporal artery, which is thick and has a blood flow higher than the capacity of the middle cerebral artery in the M4 segment, a modified Y-vessel shunt is used to achieve autonomous distribution of the superficial temporal artery blood flow (16). That is, to avoid a transient large amount of blood flow from the external carotid system into the brain after anastomosis, which leads to the risk of postoperative overperfusion and edema (17). Meanwhile, the fused superficial temporal artery branch is important for the prevention of ischemic necrosis of the scalp and the promotion of vascular neovascularization (18). In patients with poor recipient vascularization, double-branch freeing and fusion of the superficial temporal artery is also significantly more effective than single-branch fusion (19).

In our newly introduced surgical approach, isolation and protection of the vessels of the superficial temporal artery and its branches (frontal and parietal) are critical to the success of the procedure. In our group, we generally used an enlarged pterional incision and designed the surgical incision along the outer edge of the vessels according to the main stem and double branch course of the superficial temporal artery. The depth of the incision was noted where the apical branch crossed the edge of the flap, and it was important to protect the vessel from being cut off. The bone window is designed in a conventional square shape. The meningeal artery with intracranial compensation is preserved and the dura is flipped and applied to the cerebral surface (20). By flipping the temporal muscle across the gap between the superficial temporal artery frontal branch and the flap, the superficial temporal artery frontal branch can be brought to the surface of the brain tissue, providing the possibility of bypass or fusion surgery (21, 22).

There are some shortcomings of the modified bypass surgical method. (1) The realization and effect of the surgery depend on the condition of the donor and recipient vessels, for example, for patients with very poor development of the superficial temporal artery or the M3-M4 segment of the brain surface vessels, it is not possible to achieve the early improvement of the blood flow through the blood vessel direct anastomotic bypass bridge, and the patients need to wait for the formation of the fused blood vessels as well as the neovascularization of the temporal muscle of the apposition to register an improvement in blood flow. For some elderly patients, the angiogenic capacity is insufficient, and the long-term effect is unsatisfactory. (2) Currently, there is no clear detection method and precise regulation of the distribution and autonomous regulation of blood flow in the two vessels of the superficial temporal artery. At present, we use electrocoagulation to control the vessels’ diameter or increase the tortuosity of blood vessels to regulate the donor’s blood flow. (3) The modified surgical methods have been carried out for a relatively short period of time, therefore, a long-term follow-up is necessary.

## Conclusion

The improved STA-MCA bypass, which fuses two branches of the superficial temporal artery with a direct bypass and a patch, could provide blood flow to multiple cerebral ischemic areas, reduce excessive blood perfusion caused by single-vessel bypass, and ensure blood supply to the scalp, with lower complications and better clinical benefits than the traditional combined bypass.

## Data availability statement

The raw data supporting the conclusions of this article will be made available by the authors, without undue reservation.

## Ethics statement

The studies involving humans were approved by the Ethics Committee of Tongji Hospital. The studies were conducted in accordance with the local legislation and institutional requirements. Written informed consent for participation was not required from the participants or the participants’ legal guardians/next of kin in accordance with the national legislation and institutional requirements.

## Author contributions

LL: Data curation, Formal analysis, Investigation, Methodology, Project administration, Validation, Visualization, Writing – original draft, Writing – review & editing. YiH: Data curation, Investigation, Methodology, Validation, Writing – original draft. YaH: Investigation, Methodology, Writing – review & editing. YL: Investigation, Methodology, Writing – review & editing. XW: Investigation, Methodology, Writing – original draft. JC: Investigation, Methodology, Writing – original draft. XZ: Methodology, Writing – original draft. KS: Methodology, Supervision, Writing – original draft. TL: Conceptualization, Methodology, Supervision, Writing – original draft. SW: Investigation, Methodology, Writing – review & editing. CG: Writing – original draft. HZ: Writing – original draft, Writing – review & editing.
