# Supplementary material for: Clinical effect of a modified superficial temporal artery-middle cerebral artery bypass surgery in Moyamoya disease treatment
Source: Front Neurol. 2023 Oct 24;14:1273822. doi: 10.3389/fneur.2023.1273822 (PMC10628485; doi:10.3389/fneur.2023.1273822)
Supplement: Supplementary file 1 [file Table_1.DOC]

Supplementary Table 1. Postoperative outcome stratified by direct and indirect bypass separately

| Characteristic | Direct Bypass(n=34) | | P | Indirect Bypass(n=7) | | P |
| --- | --- | --- | --- | --- | --- | --- |
|  | Traditional(n=25) | Modified(n=9) |  | Traditional(n=5) | Modified(n=2) |  |
| Bypass patency during OP | 25（100.0%） | 9（100.0%） | 1.000 | 5（100.0%） | 2（100.0%） | 1.000 |
| Bypass patency at last FU | 25（100.0%） | 9（100.0%） | 1.000 | 3（60.0%） | 2（100.0%） | 1.000 |
| Matsushima grade at last FU |  |  | 0.732 |  |  | 0.571 |
| A | 11（44.0%） | 5（55.6%） |  | 0（0.0%） | 1（50.0%） |  |
| B | 13（52.0%） | 4（44.4%） |  | 2（40.0%） | 1（50.0%） |  |
| C | 1（4.0%） | 0（0.0%） |  | 3（60.0%） | 0（0.0%） |  |
| mRS score at last FU |  |  | 0.729 |  |  | 0.099 |
| 0 | 9（36.0%） | 4（44.4%） |  | 0（0.0%） | 0（0.0%） |  |
| 1 | 10（40.0%） | 2（22.2%） |  | 0（0.0%） | 2（100.0%） |  |
| 2 | 5（20.0%） | 2（22.2%） |  | 3（60.0%） | 0（0.0%） |  |
| 3 | 1（4.0%） | 1（11.1%） |  | 2（40.0%） | 0（0.0%） |  |
| 4 | 0（0.0%） | 0（0.0%） |  | 0（0.0%） | 0（0.0%） |  |

FU, follow-up; OP, operation
